# Do Peer Relations in Adolescence Influence Health in Adulthood? Peer Problems in the School Setting and the Metabolic Syndrome in Middle-Age

**DOI:** 10.1371/journal.pone.0039385

**Published:** 2012-06-27

**Authors:** Per E. Gustafsson, Urban Janlert, Töres Theorell, Hugo Westerlund, Anne Hammarström

**Affiliations:** 1 Family Medicine, Department of Public Health and Clinical Medicine, Umeå University, Umeå, Sweden; 2 Epidemiology and Global Health, Department of Public Health and Clinical Medicine, Umeå University, Umeå, Sweden; 3 Stress Research Institute, Stockholm University, Stockholm, Sweden; The University of Hong Kong, Hong Kong

## Abstract

While the importance of social relations for health has been demonstrated in childhood, adolescence and adulthood, few studies have examined the prospective importance of peer relations for adult health. The aim of this study was to examine whether peer problems in the school setting in adolescence relates to the metabolic syndrome in middle-age. Participants came from the Northern Swedish Cohort, a 27-year cohort study of school leavers (effective n = 881, 82% of the original cohort). A score of peer problems was operationalized through form teachers’ assessment of each student’s isolation and popularity among school peers at age 16 years, and the metabolic syndrome was measured by clinical measures at age 43 according to established criteria. Additional information on health, health behaviors, achievement and social circumstances were collected from teacher interviews, school records, clinical measurements and self-administered questionnaires. Logistic regression was used as the main statistical method. Results showed a dose-response relationship between peer problems in adolescence and metabolic syndrome in middle-age, corresponding to 36% higher odds for the metabolic syndrome at age 43 for each SD higher peer problems score at age 16. The association remained significant after adjustment for health, health behaviors, school adjustment or family circumstances in adolescence, and for psychological distress, health behaviors or social circumstances in adulthood. In analyses stratified by sex, the results were significant only in women after adjustment for covariates. Peer problems were significantly related to all individual components of the metabolic syndrome. These results suggest that unsuccessful adaption to the school peer group can have enduring consequences for metabolic health.

## Introduction

Social ties are important determinants of well-being and health in children, adolescents and adults. Numerous studies within life course epidemiology have also demonstrated that unfavorable social circumstances in early life may lead to enduring health risks in adulthood. Most life course research on the long-term health effects of early social circumstances has however focused on parental socioeconomic status as indicative of children’s and adolescents’ social position in the overarching societal hierarchy [1], or on adverse circumstances originating in the family setting [2], [3]. While the family certainly is an important developmental context, children and adolescents are also faced with the relational potentials and hazards of the peer group in the school milieu, a topic which has received comparatively little attention as origins of health disparities in adulthood. The present 27-year prospective cohort study specifically focuses on adolescents’ relationships with school peers as a determinant of the metabolic syndrome in middle-age.

The school class is a key setting for peer relations to form and develop, and as children grow older and reach adolescence peer relations become increasingly important sources of interaction and support, relative to the social bonds within the family. On the one hand, friendships support children and adolescents in their normative transitions across development [4], [5], and can buffer against the impact of burdensome circumstances in other spheres of life, such as poor relations with parents [6]. On the other hand, failure to form supportive relations with peers can involve threats to well-being and development [5], [7], [8]. This paper focuses on two overlapping dimensions of peer relations which signify one’s success in this arena: degree of perceived unpopularity and of social isolation, which collectively will be labeled *peer problems*. Hierarchies based on acceptance and rejection by others form in peer groups as soon as children enter the world of peers, and the popularity one is ascribed indicates one’s position within this hierarchy. Popularity is commonly divided into sociometric popularity, capturing how well liked and accepted one is by peers, and perceived popularity, which reflects one’s social influence and reputation in the peer group [9], [10]. A related aspect of peer relations is a relative lack of social interaction with peers, social isolation. Isolation from peers can be the result of the interplay between an individual being rejected by peers (‘active isolation’) or the individual isolating her−/himself from the peer group (‘social withdrawal’), e.g. due to poor self-esteem or self-perceived difficulties in social interactions [11].

To be unpopular or socially isolated have been implicated as having adverse consequences for children’s and adolescents’ mental well-being and development [11], [12]. Research in recent years also suggests that the health impact of peer relationships in childhood and adolescence might stretch across the life course. For example, low peer status and lack of friends in childhood have been shown to predict adult psychiatric diagnosis and inpatient care in the Stockholm Birth Cohort [13], [14] as well as self-reported health and self-reported long-standing illness in adulthood in the Aberdeen Children of the 1950s Cohort [15], [16]. But peer problems may also leave its marks on the body in more concrete ways. Social isolation in childhood has been prospectively linked to clustering of cardiovascular risk factors (e.g., BMI, blood pressure and lipids) in young adulthood (age 26 years) in the Dunedin Multidisciplinary Health and Development Study [17]. To the authors’ knowledge, no study has investigated whether such metabolic consequences of peer relationships remain into middle adulthood. However, a graded association has been observed between peer status in pre-adolescence and adult disease risk in the Stockholm Birth Cohort, not only for mental disorders but also for cardiovascular and diabetic disease [18]. Although this study was limited by morbidity information from register data on inpatient care, its findings lend support to the hypothesis that peer relationships might impact on metabolic disease even later in adulthood.

It has been suggested that social relations impact on bodily processes by generalized rather than specific patterns [19], and that it therefore might be sensible to examine how social relations relate to broader health outcomes. The metabolic syndrome (MetS) represents a cluster of metabolic and cardiovascular disturbances, including central obesity, dyslipidemia, high blood pressure and disturbed glucose metabolism [20]. The MetS is thought to develop gradually over the life course, and a range of social and metabolic factors in early life have been found to predict its development in adulthood, e.g. childhood and adolescent BMI, systolic blood pressure, lipids [21], [22], socioeconomic conditions [22], [23] and family environment [3], [24].

The potential pathways whereby peer relations could influence the risk of the metabolic syndrome in adulthood are manifold and include direct and indirect biological, behavioral and social pathways. Stress is proposed as one general pathway explaining how peer relations may predict future health [13]. Peer problems could possibly impact directly on metabolic health at an early stage through activation of biological stress systems [25], and early metabolic disturbances, such as excessive body mass and blood pressure, might track over the life course [26] and could thereby contribute to the development of the metabolic syndrome in adulthood. Social isolation is also thought to contribute to poor mental health in adolescence [7], [27] and adulthood [13], which can possibly influence adult obesity [28], [29] and, at least in the short-run, the progression of insulin resistance [30]. Peer relations in childhood or adolescence may also act on later health by peer socialization of health-damaging behaviors [12] and by influencing the development of the social skills necessary to successfully interact with others in later life. Moreover, peer relations are thought to exert an important but complex influence on academic achievement [31], [32], which itself is an important predictor for adult health behaviors and health [33], at least partially operating through poor socioeconomic conditions and unemployment in adulthood [34].

However, one’s ability to succeed in the peer group is also influenced by several of the putative mediators described above. For example, behavioral and emotional problems [11] and pre-existing metabolic risks such as a large body size in adolescence [35], [36] may in themselves contribute to rejection by peers. An important empirical question is therefore whether any enduring health consequences of peer relations mainly reflect covariance with concurrent risk factors.

This complex web of potential causal and non-causal relationships places high methodological demands on studies seeking to examine whether peer relations during upbringing relate to adult health. The aim of this 27-year cohort study of 881 Swedish women and men is to examine whether teacher-rated peer problems at age 16 years is related to the metabolic syndrome at age 43, while taking into account health, health behaviors and social conditions in adolescence and adulthood.

## Methods

### Ethics Statement

Ethical approval has been granted by the Regional Ethical Review Board in Umeå, Sweden. Separate written informed consent was not requested by the Ethical Review Board, as the participant is regarded as giving written consent when completing the questionnaire at each data collection wave. All participants were clearly informed that participation in the study is voluntary and that they can decide to withdraw from participation at any time, without giving any explanation.

### Participants

The present report is based on data from the Northern Swedish Cohort [37], a 27-year cohort based on all school leavers of the ninth (final) grade of the Swedish compulsory school, in the northern Swedish municipality of Luleå, at participant age 16 years (n = 1083). Follow-ups have been conducted at age 18, 21, 30 and 43 years, with n = 1010 still participating at the age of 43 years (94% of those n = 1073 still alive). The cohort has been shown to be comparable to this age cohort of Sweden with regard to socioeconomic factors, health and health behaviors [37].

The analytical sample of the present report comprises participants for whom teacher reports at age 16 and clinical measures at age 43 were available (n = 881, 423 women and 458 men), corresponding to 82% of the original cohort still alive and 87% of those participating at age 43 years.

### Procedures and Measures

At age 16 and 43 years, the participants completed a self-administered questionnaire covering social circumstances, behavioral factors and health.

When the participants were 16 years of age, each form teacher (n = 56) was interviewed using a structured interview inspired by the Rutter’s Teacher Questionnaire [38], covering items about the teachers’ assessment of each student’s behavior, well-being and functioning in the school environment as well as how the class functioned as a whole [37]. All interviews were performed by the same person, the principal investigator of the Northern Swedish Cohort (AH). Participants’ final total school grades from the ninth grade were retrieved from school records. Blood pressure (two consecutive readings) was measured in the supine position in the right arm after 10 minutes of rest, and information on weight and height measured by the school nurses as part of a compulsory school health examination was retrieved from school health records.

At age 43, the participants took part in a more comprehensive health examination at their health care center, see [22] for details. Blood pressure (two consecutive readings) and waist circumference were measured according to the WHO MONICA manual [39]. Blood samples were collected after an overnight fast and analyzed with respect to high-density lipoprotein (HDL) cholesterol, triglycerides, and glucose. Laboratory assays were performed at the Department of Clinical Chemistry, Umeå University Hospital.

#### Exposure: peer problems in the school setting at age 16 years

Peer problems were measured by two items of the form teacher interview. The teachers were asked to rate each student on two dimensions of ‘*Tendency to isolation’* – ‘*Extroversion’* and ‘*Unpopularity among peers’* – *‘Popularity among peers’*, respectively, on a discrete scale from 1 (most isolated/unpopular) to 6 (most extroverted/popular). As the two items were strongly related (r = .65) and exploratory analyses suggested similar association to adult health (data not shown), the two items were combined into a single measure rather than examined as separate exposures. The scores were reversed to form two separate scores of Isolation and Unpopularity (range 0–5, higher score representing more isolation and unpopularity, respectively) and added together (range 0–10, internal consistency estimated at Cronbach’s α = .79) to form an index of peer problems. The index was z-transformed (separately for women and men) to aid the interpretation of the logistic regression estimates.

#### Outcome: the metabolic syndrome at age 43 years

The metabolic syndrome was defined according to the International Diabetes Federation’s criteria [40]: A) Waist circumference ≥80 cm for women/≥94 cm for men, and B) Two or more of the following four criteria: 1. Raised triglycerides (≥1.7 mmol/L) or specific treatment for that lipid abnormality, 2. Reduced HDL cholesterol (<1.29 mmol/L for women/<1.03 mmol/L for men) or specific treatment for that lipid abnormality, 3. Raised blood pressure (SBP≥130 mmHg or DBP≥85 mmHg) or treatment of hypertension, 4. Raised fasting glucose (≥5.6 mmol/L) or diagnosed type 2 diabetes. Sixty-one participants were treated for hypertension while no participants were treated specifically for either hypertriglyceridemia or low HDL cholesterol. Current type 2 diabetes (n = 17) was based on self-reported presence of diabetes at age 43, excluding those who also reported diabetes at age 30 (n = 4, all of whom also reported diabetes at age 21), who were regarded as having type 1 diabetes.

#### School adjustment in adolescence

Final total (mean) *school grade* of the ninth grade, at participant age 16, was recoded according to deciles (range 1–10), with those with no grade coded as the lowest decile.

Self-reported *contentment with school lessons*, *contentment with classmates*, and *contentment with other time in school* were all measured by self-administered questionnaires at age 16, with responses on five-level Likert scales (‘very good’ = 1, ‘good’ = 2, ‘neither particularly good or particularly bad’ = 3, ‘bad’ = 4, ‘very bad’ = 5).

#### Family conditions in adolescence

Family conditions in adolescence were operationalized through items of the self-administered questionnaire completed by the participants at age 16.

*Cumulative adversity in adolescence* was operationalized as the count of six adversities reported at age 16 years, see [2] for details. The counted adversities were *residential crowding* ( = not having a own room), *residential mobility* ( = having moved more than three times during one’s lifetime, corresponding to the 80^th^ percentile), *parental unemployment* ( = either parent being unemployed), *parental illness* ( = either parent suffering from physical or mental illness, or having alcohol problems), *parental separation or loss* ( = parents being divorced, or either parent deceased) and *material standard of living* ( = having less than four (80^th^ percentile) items in the family’s possession, from a list of eleven items, e.g. car and color TV).

The participants also responded to a questionnaire item about perceived quality of *contact with mother* and *contact with father,* with responses on a five-level Likert scale (‘very good’ = 1, ‘good’ = 2, ‘neither particularly good or particularly bad’ = 3, ‘bad’ = 4, ‘very bad’ = 5). Those who did not respond to the question but were bereaved of their mother (n = 2) or father (n = 16) were coded as 5 on the respective item.

#### Health in adolescence

At age 16, *body mass index* (BMI) was calculated as kg/m^2^ and *systolic* and *diastolic blood pressure* (SBP and DBP, mmHg) was operationalized as the mean of two readings.

*Aggression* was assessed by form teachers through the teacher interview, with each student’s aggression rated on a scale from 1 to 6.

#### Psychological distress in adolescence and adulthood

At age 16 and 43 years, psychological distress was operationalized as self-reported frequency of *sleep problems*, *nervous problems* and *depressive problems* during the preceding 12 months, with response on four-level Likert scales (‘never’ = 1, ‘now and then’ = 2, ‘often’ = 3. ‘all the time’ = 4). Due to low internal consistency at age 16 (Cronbach’s α = .64) the measures were treated as separate variables in the analysis, whereas the items were added together at age 43 (range 3–12, Cronbach’s α = .77).

#### Socioeconomic disadvantage in adolescence and adulthood

*Socioeconomic disadvantage in adolescence* was based maternal and paternal occupation, coded according to Statistics Sweden’s classification [41] and dichotomized into 1 = both parents being manual workers and 0 = one or both parents being non-manual employees or self-employed [22]. *Socioeconomic disadvantage in adulthood* was based on own occupation as reported in the questionnaires completed at age 43 years, and similarly dichotomized into 1 = manual worker and 0 = non-manual employee or self-employed.

#### Health behaviors in adolescence and adulthood

Health behaviors were assessed through the self-administered questionnaire at age 16 and age 43 years.

Daily *smoking* and *snuff use* were coded as yes = 1, no = 0. Frequency of *physical activity* was rated on a six-level Likert scale (‘daily’ = 6, ‘several times a week’ = 5, ‘about once a week’ = 4, ‘several times a month’ = 3, ‘about once a month’ = 2, ‘seldom/never’ = 1) and frequency of c*onsumption of sweets* on a five-level Likert scale (‘several times a day’ = 5, ‘about once a day’ = 4, ‘several times a week’ = 3, ‘about once a week’ = 2, ‘less frequent than once a week’ = 5). Yearly *alcohol consumption* was estimated from reported frequency and volume of beverages typically consumed, and categorized into quintiles (range 1–5, with quintile 1 and 2 collapsed at age 16 due to high frequency of non-drinkers). At age 43 years, frequency of *consumption of fruits and berries* and of *consumption of vegetables* were also inquired about, on seven-level Likert scales (‘three times a day or more often’ = 7, ‘twice a day’ = 6, ‘once a day’ = 5, ‘5–6 times a week’ = 4, ‘3–4 times a week’ = 3, 1–2 times a week’ = 2, ‘a few times a month or never’ = 1).

#### Adult social circumstances

All indicators of adult social circumstances were based on the self-administered questionnaire at age 43.

*Unemployment* was based on an item about current labor market situation, coded as current unemployment, in labor market program or on disability pension ( = 1) versus currently employed or studying ( = 0).

An item about cohabitation defined *single marital status* ( = 1), versus married/cohabiting ( = 0).

*Social support* was operationalized by Availability of Social Integration (AVSI, the sum of six four-level Likert items with range 6–20) and Availability of Attachment (AVAT, four six-level Likert items with range 4–24) from the Interview Schedule for Social Interaction (ISSI) [42].

### Statistical Analysis

As previous research has suggested that the long-term impact of peer relations may act on adult health through different pathways for women and men [15], [16], the main analyses were performed both on the entire sample and stratified by sex. The analytical sample comprised 881 participants (423 women and 458 men). To maximize power and to mitigate the risk for potential selection bias, all available data was used for each analysis. Due to item non-response on specific covariates the effective n is lower in adjusted analyses (lowest n = 848, 412 women and 436 men).

The analytical sample size was constrained by participants not completing the clinical measures necessary to operationalize the MetS at age 43 years. To examine to which degree the drop-out was systematic, the analytical sample was compared to those excluded due to non-completion of clinical measures, with respect to all independent variables considered (χ^2^, Mann-Whitney U, or t test). Excluded women had more peer problems (t test, p = .006) and higher SBP (t test, p = .011) at age 16 and reported more psychological distressed at age 43 (Mann-Whitney U test, p = .037), whereas excluded men had lower final school grades (t test, p = .001) and reported poorer contact with mother (Mann-Whitney U test, p = .013) at age 16 years. No other significant differences were found for any of the other variables (results not shown; see Table 1 for a full list of the variables).

**Table 1:** Descriptive statistics of covariates by quartiles of peer problems at age 16 years.

Variable group	Variable	Quartiles of peer problems at age 16				p value
		Q1	Q2	Q3	Q4	
	Sex, % women	36	65	50	43	.399^a^
Adolescent Health	Body Mass Index (kg/m^2^), M(SD)	19.8(2.4)	20.0(2.7)	19.9(2.7)	19.8(2.9)	.590^b^
	Systolic BP (mmHg), M(SD)	122(13.5)	120(12.7)	121(13.5)	121(13.2)	.655 b
	Diastolic BP (mmHg), M(SD)	69.2(10.2)	67.8(10.9)	68.7(11.8)	69.6(11.1)	.263 b
	Aggression, M(SD)	1.54(1.35)	1.73(1.21)	1.82(1.17)	1.94(1.42)	.001 b
	Sleep problems, % reporting any sleep problems	29	36	39	41	.002 b
	Nervous problems, % reporting any nervous problems	22	33	37	30	.158 b
	Depressive problems, % reporting any depressive problems	71	74	76	72	.366 b
Adolescent health behaviors	Smoking, %	20	29	27	29	.070 a
	Snuff, %	6.5	7.5	8.5	6.3	.896 a
	Alcohol consumptions, % in quintile 5	16	19	21	22	.517 a
	Physical activity, % responding ‘daily’	15	7.5	7.4	6.3	<.001 b
	Consumption of sweets, % responding ‘several times a day’	2.3	2.7	5.1	1.5	.066 a
Adolescent school adjustment	Final school grade (deciles), M(SD)	6.7(2.6)	6.2(2.7)	5.5(2.8)	4.0(2.7)	<.001 b
	Contentment with lessons, % responding worse than ‘good’	51	60	58	66	.003 b
	Contentment with classmates, % responding worse than ‘good’	11	12	16	26	<.001 b
	Contentment with school, % responding worse than ‘good’	39	39	48	48	.002 b
Adolescent family conditions	Socioeconomic disadvantage, % with manual worker parents	24	37	45	46	<.001 a
	Cumulative adversity, % reporting one or more adversity	57	66	69	77	<.001 a
	Contact with mother, % responding worse than ‘very good’	32	36	37	38	.139 b
	Contact with father, % responding worse than ‘very good’	46	60	57	48	.315 b
Adult psychological distress	Psychological distress, M(SD)	4.4(1.5)	4.4(1.5)	5.0(1.9)	5.0(2.0)	<.001 b
Adult health behaviors	Smoking, %	15	16	22	28	<.001 a
	Snuff, %	21	23	20	24	.758 a
	Alcohol consumption, % in quintile 5	27	17	16	15	.002 a
	Physical activity, % responding ‘daily’	5.1	12	8.5	12	.145 b
	Consumption of sweets, % responding ‘several times a day’	0.9	2.1	0.7	2.9	.136 b
	Fruit consumption, M(SD)	4.3(1.8)	4.7(1.5)	4.4(1.7)	4.0(1.8)	.127 b
	Vegetables consumption, M(SD)	4.8(1.5)	5.0(1.4)	4.7(1.5)	4.6(1.7)	.076 b
Adult social circumstances	Unemployment	5.2	4.9	10	10	.011 a
	Socioeconomic disadvantage, % manual workers	22	29	34	56	<.001 a
	Single marital status, % single/not cohabiting	19	19	22	34	<.001 a
	Availability of attachment, M(SD)	15.9(4.4)	15.1(3.7)	14.7(4.2)	14.1(4.1)	<.001 b
	Availability of social integration (AVSI), M(SD)	9.8(2.5)	9.3(2.3)	9.7(2.4)	10.0(2.7)	.193 b

Note that for ordinal variables ≤5 levels, descriptive statistics are only displayed for collapsed response levels, as indicated in the variable column, whereas bivariate associations were estimated using the full range of each variable.

a p value from Mantel-Haenszel test (dichotomous variables, or ordinal variables ≤5 levels with <20% cells with expected frequencies <5).

b p value from Spearman’s rho (continuous variables, or ordinal variable ≥6 levels, or ≤5 levels with >20% cells with expected frequencies <5).

The aim of this study was to examine whether peer relations in school were related to adult health risk. However, as peer problems were rated by the same form teacher for all students in the same school class, the ratings were not strictly independent observations, which could bias the estimates from individual-level analysis. To examine whether clustering of data by teacher/class influenced the estimates, the intraclass coefficient (ICC) for teacher/class and the change of estimates due to choice of analytical method were evaluated. As the ICC is difficult to interpret for dichotomous outcomes [43] an alternative MetS variable comprising the sum of the five diagnostic criteria fulfilled (range 0–5) was used as the outcome in Linear Mixed Models. In a null model containing only the level 2 clusters by teacher/class, the ICC was estimated to 0.01 and the design effect to 1.25 (calculated as deff  = 1+ (mean cluster size −1)×ICC [44], mean cluster size  = 19). Adding the individual covariate peer problems, the fixed effect (SE) was estimated at 0.227 (0.0476), p<.001, whereas the corresponding numbers from simple OLS regression was 0.224 (0.0470), p<.001. The low ICC and design effect [44] in combination with the numerically and stochastically nearly identical estimates in analyses with and without consideration of clustering, suggested that individual-level analyses would not lead to biased inferences. Thus, individual-level methods where used in subsequent analyses to address our research question.

The main analyses consisted of a series of logistic regression models, with MetS at age 43 regressed on z-transformed peer problems at age 16 (Model 0 = crude model), with subsequent addition of covariates that theoretically could explain the association. To be able to disentangle the explanatory value of particular pathways and to avoid biased estimates due to too few events per variable [45], sets of covariates measuring conceptually related factors were added to the crude model in separate models, rather than including all covariates in the same model. The separate models included adjustment for adolescent health (Model 1: BMI, systolic blood pressure, diastolic blood pressure, self-reported sleep, nervous and depressive problems, teacher-rated aggression at age 16), health behaviors (Model 2: self-reported alcohol consumption, smoking, snuff use, physical activity and consumption of sweets at age 16), school adjustment (Model 3: final school grade, self-reported contentment with school lessons, with other time in school and with classmates at age 16) and family conditions (Model 4: cumulative adversity, perceived contact with mother, perceived contact with father, and socioeconomic disadvantage at age 16), and for adult psychological distress (Model 5: self-reported psychological distress at age 43), health behaviors (Model 6: self-reported alcohol consumption, smoking, snuff use, physical activity, and consumption of sweets, fruits and vegetables at age 43), and social circumstances (Model 7: Self-reported unemployment, socioeconomic disadvantage at age 43, single marital status, availability of attachment (AVAT), availability of social interaction (AVSI)). Although we aimed at limiting the number of covariates in the analyses, there is also a possibility that the additive effect of health-related, behavioral or social chains of risk over the life course [46], rather than conditions in either adolescence or adulthood, explained the main association. Complementary analyses were therefore conducted with simultaneous adjustment for covariates in adolescence and adulthood.

## Results

As can be seen in Table 1, degree of peer problems was related to a range of unfavorable health, health behaviors and social conditions in adolescence as well as in adulthood. Peer problems were positively related to more teacher-rated aggression and self-reported sleep problems in adolescence but not to early cardiovascular risk factors (BMI and systolic blood pressure). Adolescents with more peer problems engaged less frequently in physical activity and tended to smoke more frequently. Pertaining to school adjustment, adolescents with more peer problems achieved lower final school grades and reported higher discontentment with school lessons, classmates and with other time in school, and they were also socioeconomically disadvantaged to a greater degree and were exposed to more accumulated adversity. In adulthood, those with past experience of peer problems were more distressed, smoked more, and tended to eat vegetables less frequently, but consumed less alcohol. Socially, they were at risk for unemployment, socioeconomic disadvantage, living alone and for having a more limited social network (as indicated by AVAT). In summary, bivariate analyses generally indicated that peer problems in adolescence involved a multitude of potentially health-damaging health, behavioral and social conditions in both adolescence and middle-age.

The point prevalence of MetS was estimated at 27.1% (19.1% in women and 34.5% in men). Table 2 shows odds ratios from logistic regression analyses with MetS at age 43 regressed on (z-transformed) peer problems at age 16, with the subsequent addition of covariates (covariate estimates not shown). In the total sample, the crude model (Model 0) indicated that one higher SD of peer problems corresponded to 36% higher odds for developing MetS at age 43. In multiple models, adjustment for adolescent health (Model 1) and adolescent health behaviors (Model 2) lead to a small to moderate attenuation of the OR for peer problems. Except for sex [Model 1: OR (95% CI) = 0.90 (0.81–1.01)], BMI [Model 1: OR (95% CI) = 2.07 (1.45–2.97)] and physical activity [Model 2: OR (95% CI) = 0.87 (0.77–0.97)] were the only significant covariates in these models. Adjustment for factors indicating how well the participants adapted to the school environment (Model 3) lead to a more substantial OR attenuation for peer problems, although the estimate remained significant. Of the school adjustment factors, final school grade was the only significant covariate [OR (95% CI) = 0.91(0.85–0.97)]. Adjustment for adolescent family conditions (Model 4) lead to a meager OR attenuation, and perceived contact with father was the only significant covariate [OR (95% CI) = 1.16 (1.00–1.35)]. In models adjusting for factors at age 43 (see Table 2, Model 5: psychological distress, Model 6: health behaviors, and Model 7: social circumstances) the OR for peer problems was slightly to moderately attenuated. In these models, psychological distress [Model 5: OR (95% CI) = 1.11 (1.02–1.21)] and physical activity [Model 6: OR (95% CI) = 0.85 (0.77–0.94)] were the only significant covariates, with borderline significant estimates for fruit consumption [Model 6: OR (95% CI) = 0.90 (0.81–1.01)] and socioeconomic disadvantage [Model 7: OR (95% CI) = 1.39 (0.99–1.96)].

**Table 2:** Logistic regression analyses with metabolic syndrome at age 43 regressed on peer problems at age 16.

Covariates included	Total sample			Women			Men		
	n	OR	95% CI	n	OR	95% CI	n	OR	95% CI
Model 0: Crude	881	1.36	1.17–1.58	423	1.59	1.25–2.02	458	1.22	1.01–1.49
Model 1: Adolescent health^a^	869	1.34	1.14–1.58	418	1.58	1.23–2.03	451	1.18	0.95–1.45
Model 2: Adolescent health behaviors^b^	870	1.29	1.10–1.52	417	1.53	1.19–1.96	453	1.15	0.93–1.42
Model 3: Adolescent school adjustment^c^	878	1.23	1.04–1.46	422	1.44	1.10–1.87	456	1.12	0.90–1.39
Model 4: Adolescent family conditions^d^	874	1.33	1.13–1.56	420	1.53	1.19–1.97	454	1.23	1.00–1.51
Model 5: Adult psychological distress^e^	878	1.33	1.14–1.55	422	1.55	1.21–1.98	456	1.20	0.98–1.46
Model 6: Adult health behaviors^f^	862	1.33	1.14–1.57	417	1.52	1.18–1.96	445	1.23	0.99–1.52
Model 7: Adult social circumstances^g^	848	1.31	1.11–1.55	412	1.47	1.13–1.90	436	1.21	0.98–1.51

The following covariates were included (estimates not shown), in addition to z-transformed peer problems (all models) and sex (total sample models only):

a BMI, systolic blood pressure, diastolic blood pressure, self-reported sleep, nervous and depressive problems, teacher-rated aggression at age 16.

b Self-reported alcohol consumption, smoking, snuff use, physical activity and consumption of sweets at age 16.

c Final school grade from school records, self-reported contentment with school lessons, contentment with other time in school and contentment with classmates at age16.

d Self-reported cumulative family adversity, contact with mother, contact with father, socioeconomic disadvantage at age 16.

e Self-reported psychological distress at age 43 (sum of sleep, nervous and depressive symptoms).

f Self-reported alcohol consumption, smoking, snuff use, physical activity, and consumption of sweets, fruits and vegetables at age 43.

g Self-reported unemployment, single marital status, availability of attachment (AVAT), availability of social interaction (AVSI), socioeconomic disadvantage at age 43.

**Figure 1:**
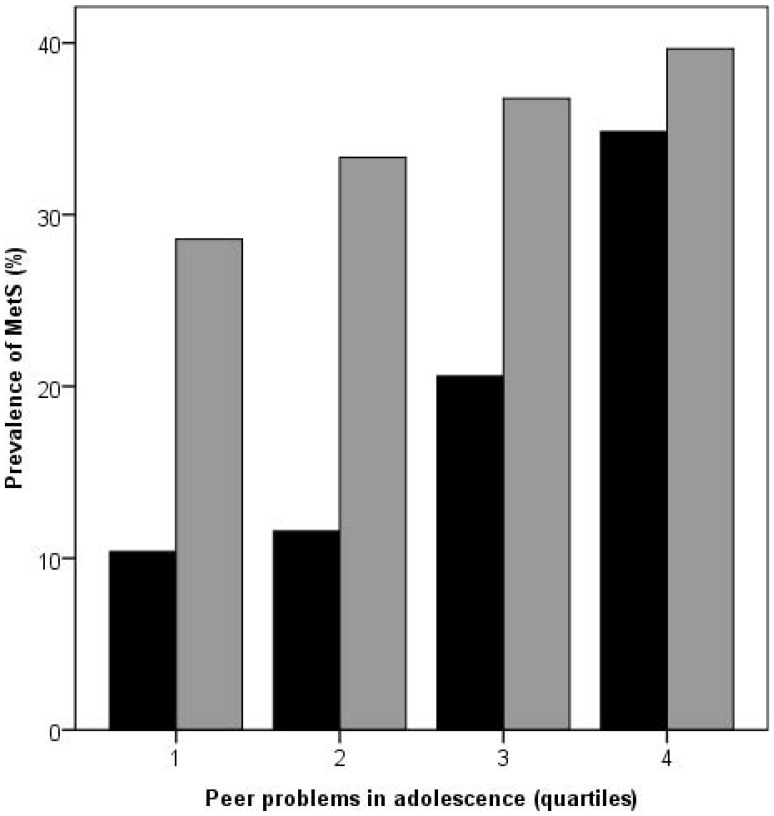
Adult metabolic syndrome (MetS) by adolescent peer problems in women (black) and men (grey).

Corresponding analyses stratified by sex (Table 2) suggested similar associations as in the total sample, but with a numerically higher OR in women than in men, corresponding to 59% higher odds for the MetS for each SD increase in peer problems in women, and 22% higher odds in men. The interaction between peer problems and sex was however not significant (p = .100, data not shown). Graphical depiction of the association (Figure 1) suggested that there was a dose-response relationship between peer problems and MetS in both women and men. Adjusting for covariates (Table 2, Model 1–7) lead to comparable magnitude of OR attenuation in women and men as in the total sample, although the OR for men was reduced below significance by any set of adjustment whereas the OR in women remained significant throughout the models.

To examine whether discrete health-related, behavioral or social life course pathways of risk, rather than factors at a specific life course period, would explain the association, complementary analyses were done with simultaneous adjustments for adolescent and adult covariates in the total sample (data not shown). As the number of covariates in several of these combined models grew exceedingly large, they should be regarded as explorative analyses. Results showed little additional attenuation of the main association in models with simultaneous adjustment for adolescent health and adult psychological distress [Model 1+5 covariates: OR (95% CI) = 1.31 (1.11–1.54), n = 867], for adolescent and adult health behaviors [Model 2+6 covariates: OR (CI) = 1.30 (1.09–1.53), n = 851], for adolescent school adjustment and adult social circumstances [Model 3+7 covariates: OR (CI) = 1.25 (1.05–1.50), n = 845], for adolescent family conditions and adult social circumstances [Model 4+7 covariates: OR (CI) = 1.30 (1.10–1.54), n = 841], or for all covariates [Model 1–7 covariates: OR (CI) = 1.24 (1.01–1.53), n = 798]. Thus, these explorative analyses suggested that the combined effect of factors in adolescence and adulthood did not fully explain the association between peer problems and MetS.

To explore whether the association between peer problems and MetS was driven by associations to particular components of the MetS, complementary simple logistic regression analyses where performed with each component of the MetS regressed on peer problems (data not shown). Results showed that peer problems were related to high waist circumference [OR (95% CI) = 1.18 (1.03–1.36), n = 887] high triglycerides [1.29 (1.11–1.50), n = 891], low HDL cholesterol [1.32 (1.14–1.53), n = 888], high blood pressure [1.26 (1.10–1.43), n = 947], and high fasting glucose [1.21 (1.02–1.42), n = 910]. Thus, peer problems in adolescence were related to all individual components of the MetS at age 43, albeit slightly more weakly than to the MetS itself.

## Discussion

The main finding of this study is that the degree of peer problems in adolescence, as assessed by form teachers, is related to a higher risk of the metabolic syndrome in middle age, independently of a range of potentially health-damaging factors in adolescence and adulthood. Our results corroborate the general notion that peer relationships in childhood or adolescence may impact on adult health, and expand current knowledge by suggesting that early peer problems can impact on multiple metabolic systems as late as in middle age, independently of other risk factors.

Despite the considerable methodological disparities between the present study and previous studies (see Methodological Considerations), our findings show remarkable similarities to results from previous studies on a number of points: that the association between peer problems and adult health is to a large degree independent of a range of putative mediators, that it is fairly graded, and that it is potentially stronger in women than in men. These points will be discussed in order.

First, the association between peer problems and adult metabolic syndrome was only partially explained by the suggested mediators or confounders, despite the wide range of adverse social, behavioral and health-related factors in both adolescence and adulthood related to peer problems in adolescence. Neither set of conceptually related covariates attenuated the association substantially. Of the factors examined, adjustment to the school environment in general, and particularly academic achievement (measured by final school grade), emerged as the most important pathway. A prominent explanatory role of academic achievement has also been suggested in previous research [14]. Previous studies have also found that peer status relates to specific morbidities independently of childhood social class [18], and to self-reported health in adulthood independently of family circumstances, cognitive status and behavioral problems in childhood, social relations and health behaviors in adulthood, and in women also of adult socioeconomic circumstances [15], [16]. Moreover, social isolation in childhood has been linked to clustering of cardiovascular risk factors in young adulthood independently of childhood and adult SES, childhood IQ and overweight and adult health behaviors [17]. This leaves the question about possible mechanisms empirically unanswered. With specific regard to MetS, complementary analyses suggested that peer problems were related to all individual components of MetS, which suggests a broad and nonspecific impact on biological systems, in accordance with the proposed indiscriminatory impact of social ties on physiological processes [19]. Indeed, we have previously reported that social adversity around the transition into adulthood is related to cumulative biological risk in adulthood [2]. One potential pathway whereby social relationships could lead to such wide-ranging physiological effects is activation of physiological stress systems, which are highly sensitive to social challenges [47] and thought to contribute to cumulative dysregulations across multiple physiological systems [48], and in the long run, to the metabolic syndrome [25]. It is noteworthy that, in the present data, self-reported contentment with school, including contentment with classmates, did not appear to explain the main association. We do not know whether this is simply an indication of measurement error or whether contentment with peers does not cover the relevant component by which peer problems act on health (e.g., loneliness [49], [50]). Alternatively, given that stressors are known to elicit a physiological response irrespective of subjective distress or negative affect [51], it is also possible that the association is mediated by pathways not easy to consciously appraise. It is also possible that other socially mediated pathways are important in explaining the life course association between peer relations and adult health in general and the metabolic syndrome in particular.

Second, our results support the notion that aspects of peer relationships are not only related to future health in the extreme end of the spectrum, e.g. restricted to those exposed to bullying or peer victimization, but that one’s difficulties with peers are represented by a health gradient in adulthood. Such a dose-response association between peer relations and adult health has been suggested previously for adult inpatient care and a range of specific morbidities [18], self-reported health [15], [16], as well as for cumulative cardiovascular risk in young adulthood [17]. However, one study reports a gradual increase of risk for anxiety or depression disorder for women across accepted, peripheral and marginalized peer status in childhood, with no difference between accepted and popular past peer status [13]. This general pattern is consistent with the graphical pattern depicted in Figure 1 among women, i.e. that the gradient is steeper in women with more peer problems. Nevertheless, it is noticeable that overall, this gradient still appears to be relevant over the greater part of the spectrum of peer problems.

Third, we found that the association between peer problems and adult health was numerically stronger in women than in men, although this difference did not reach statistical significance. Previous studies have reached different conclusions on this point but with a preponderance for stronger results in women: numerically more pronounced in women for self-reported health and longstanding illness [15], [16], only demonstrable in women with respect to anxiety and depressive disorders [13] and demonstrable and numerically comparable in women and men for disease-specific morbidity, but differing for specific diseases [18] (note: the study by Caspi et al. [17] did not stratify analyses by sex). Although an exposure of a different nature, it should be noted that socioeconomic status in childhood and adolescence has been found to be more strongly related to adult obesity and MetS in women than in men, both in the present cohort [22], [52] and in other studies [23], [53], [54]. This health disparity is partially explained by a social chain of risk spanning over the life course [24]. It is possible that the future health risk resulting from peer problems involve partially different life course pathways for women and men. This is suggested by previous studies showing that socioeconomic circumstances largely explain the association in men only [15], [16]. Nevertheless, in the present study the association was in the same direction for both women and men, and the numerical difference in strength of association should not be overstated.

### Methodological Considerations

The main strengths of the present study are the long follow-up period, a sample rather representative of this age cohort of Sweden with comparatively low attrition rate, and the comprehensive sets of covariates we were able to include in the analyses. There are also a number of limitations which should to be highlighted.

As the analysis of the non-participants suggested (see Statistical Analysis) that women not completing the clinical measures had more peer problems in adolescence, participation bias would be expected to contribute to under- rather than overestimation of the association, in addition to imprecision of the estimates in women. Excluded women also reported slightly more distress in adulthood and had higher systolic blood pressure in adolescence, and excluded men had lower school grades and reported poorer contact with mother. Ultimately, the consequences of these indications of participation bias are unknown. It should be emphasized that the excellent overall retention rate made it possible to evaluate the nature of the drop-out, and the analytical sample did not differ from the internal drop-out on the majority of health, behavioral and social variables considered in the analyses. This overall similarity between the participants and non-participants on the range of variables examined suggests that those dropping out do not represent a substantially different group from those included in the analysis.

Most studies on peer relations and adult health have employed sociometric peer nominations in school-aged children [14], [15], [16], [18], whereas the present study used teacher-rated measures of perceived popularity and isolation in adolescence. As peer relations was measured at the end of the three-year Swedish high school (grade 7–9), the form teachers could be expected to be sufficiently familiar with the students. Other teacher-rated measures of peer problems [55] and popularity [56] have been found to have acceptable metric properties, but it is not readily apparent to which degree teachers’ ratings correspond to sociometric nominations or peer-perceived popularity, although some studies report acceptable agreement [56], [57]. Specifically, the consistently positive relationships between peer problems and other social risk factors (Table 1) do indeed suggest that teachers tend to identify well-adjusted rather than poorly-adjusted adolescents as popular, despite that both groups can be identified as popular by peers (cf. [57]). Research has furthermore demonstrated that popularity [58], [59] and social isolation [11] are multifaceted concepts, that they are at least partially distinct from each other [60] and, most importantly, that isolation and popularity may have different consequences for adult health [14]. Our measure of peer problems did not distinguish between any of these potentially important dimensions, which might reflect the crudeness of teacher-informant measures of peer relations in general, or of the specific measures used. Moreover, development-dependent changes of the peer group from childhood to adolescence [61] limits comparisons of peer relations measured during different developmental periods. For example, popularity [9], [62] and social isolation [11] only display moderate stability from childhood to middle adolescence. During this transition, the concurrent agreement between perceived and sociometric popularity markedly decreases, particularly in girls [9], [62], and the behavioral determinants of perceived popularity changes [9], [10], [62]. Our measure of peer problems in adolescence should therefore be interpreted as a broad concept, while we acknowledge that other specific aspects of peer relations might have importance for adult health.

Examining associations spanning over such extended time periods as in the present study –particularly when both exposures and outcome indisputably are related, causally and non-causally, to a range of external factors – makes it difficult to ascertain causality. Intelligence, for example, is one possible confounder which we did not have any data on. However, intelligence is thought to impact on later health mainly through employment conditions in adulthood [63], [64], which were included in our analyses without substantially affecting the estimates. Moreover, academic achievement has been found to be more important than intelligence in explaining the association between peer relations and adult health [14]. Still, the effect of other potential confounders cannot be ruled out.

### Conclusions

This study demonstrates that adolescents’ peer problems in the school setting relate to an increased risk of developing the metabolic syndrome in middle age, in a dose-response fashion. The association was only to a limited degree explained by other social, school, health and lifestyle factors in adolescence and adulthood. These findings emphasize the enduring developmental importance of successful adaptation to the challenges of peer interaction, and suggest that greater attention should be paid to the creation of climates fostering positive relations among students. Future research should be directed at investigating possible mechanisms whereby peer relations may contribute to later metabolic disturbances.
